# Association between post-transplant uric acid level and renal allograft fibrosis: Analysis using Banff pathologic scores from renal biopsies

**DOI:** 10.1038/s41598-018-29948-9

**Published:** 2018-08-02

**Authors:** Deok Gie Kim, Beom Seok Kim, Hoon Young Choi, Beom Jin Lim, Kyu Ha Huh, Myoung Soo Kim, Hyeon Joo Jeong, Yu Seun Kim

**Affiliations:** 1 Department of Transplantation Surgery, Severance Hospital, Yonsei University Health System, Seoul, South Korea; 2 Department of Internal Medicine (Nephrology), Yonsei University College of Medicine, Seoul, South Korea; 3 Department of Pathology, Yonsei University College of Medicine, Seoul, South Korea; 4 Department of Surgery, Yonsei University College of Medicine, Seoul, South Korea; 5 The Research Institute for Transplantation, Yonsei University College of Medicine, Seoul, South Korea

## Abstract

Several experimental studies implicate uric acid in renal injury and fibrosis. The objective of this study was to examine the association between uric acid level and allograft fibrosis after kidney transplantation. 241 adult patients who underwent kidney transplantation between 2003 and 2014 were divided into three groups according to the sex specific tertiles of mean uric acid level within the first post-transplant year. The renal biopsies performed during 1 to 5 post-transplant year were analyzed to compare the degree of interstitial fibrosis and tubular atrophy (IF/TA). Mean interval between kidney transplantation and biopsy was similar between groups (23.7 ± 15.3 vs. 30.0 ± 18.6 vs. 27.5 ± 18.5 months, *P* = 0.072). The higher tertile uric acid level was, the more advanced grade of IF/TA was shown (*P* = 0.001). Multivariate analysis identified uric acid tertile was independent risk factor for severe IF/TA (odds ratio [95% confidence interval] was 3.16 [1.13–8.82] for tertile 2 and 3.70 [1.25–10.93] for tertile 3, versus tertile 1, respectively). Other independent factors were estimated glomerular filtration rate at 1year post-transplant (0.80 [CI 0.65–0.98]) and biopsy-proven rejection (2.34 [1.05–5.21]). Graft survival over 10 years was significantly lower in tertile 3 (*P* = 0.041). The results showed that higher uric acid level after kidney transplantation was associated with more severe IF/TA.

## Introduction

Outcomes after kidney transplantation (KT) have improved over the years, but the proportion of long-term graft failures remains high^1,2^. Hyperuricemia has been reported as one of the modifiable factors predictive of long-term allograft outcomes^3–8^, although several studies have failed to identify uric acid as an independent risk factor for graft loss^9–11^. These conflicting findings are difficult to reconcile because uric acid is mainly excreted via the kidney, so graft function will affect uric acid levels^12^, and most previous studies focused on estimated glomerular filtration rate (eGFR) or loss of graft as an endpoint.

Reported mechanisms for renal injury from hyperuricemia include inhibition of endothelial nitric oxide^13^ and activation of the renin angiotensin system^14^, inducing vasoconstriction^15^, as well as vascular smooth muscle cell proliferation^16^. These changes lead to progressive renal fibrosis^17,18^, which is a common histologic finding of failing grafts^19^. In the Banff criteria, allograft fibrosis is defined as interstitial fibrosis and tubular atrophy (IF/TA) and graded according to the extent of the affected area^20^. IF/TA is associated with progression of graft dysfunction and graft failure, regardless of the underlying pathologic diagnosis^21–23^. Various causes of IF/TA are well described in literature, including rejection, hypertension, calcineurin inhibitor (CNI) toxicity, infections, and other donor or transplant factors^24–26^.

Despite the aforementioned experimental evidence, no study has heretofore investigated the pathologic changes occurring with hyperuricemia after KT, and the association between uric acid and allograft fibrosis has not yet been established in the clinical setting. In this study, our aim was to examine the association between uric acid level and allograft fibrosis after kidney transplantation.

## Results

### Baseline characteristics

**Figure 1:**
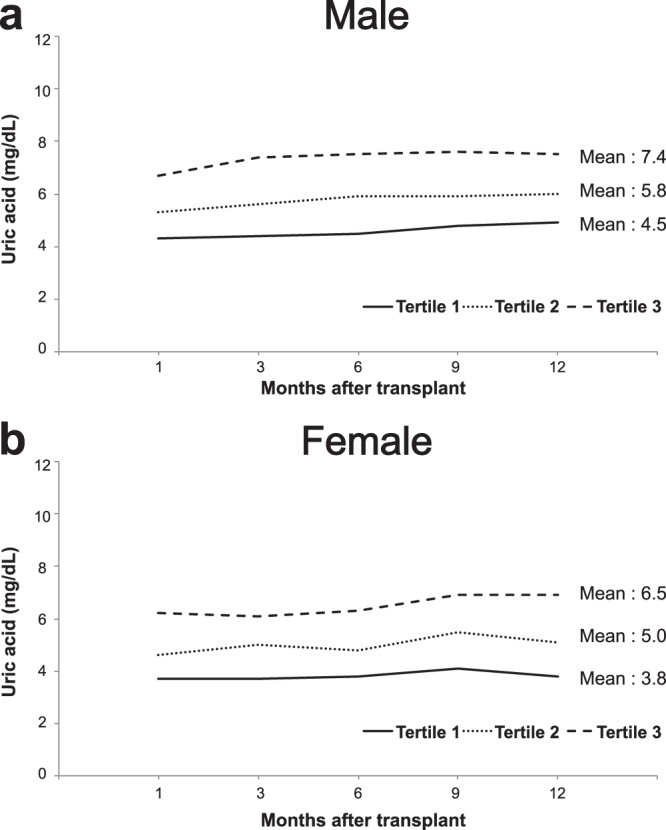
Uric acid changes within the first year after transplantation. The graphs show uric acid changes of each tertile group in males (**a**) and females (**b**) separately.

Each group of the sex specific uric acid tertile included 80 (tertile 1), 81 (tertile 2) and 80 patients (tertil 3). Changes in uric acid level over time during the first post-transplant year were demonstrated in Fig. 1. Males showed higher mean uric acid values than females for each tertile. Baseline characteristics of three groups are demonstrated in Table 1. The intervals between KT and biopsy were similar (23.7 ± 15.3 vs. 30.0 ± 18.6 vs. 27.5 ± 18.5 months, respectively; *P* = 0.072), as were the recipient’s sex, donor’s sex, retransplantation, tacrolimus use, blood pressure at 1 year, use of angiotensin converting enzyme inhibitor, use of uric acid lowering agent. However, recipient age, the body mass index, the proportions of deceased donors, donor’s age, pretransplant diabetes, duration of pretransplant dialysis and delayed graft function were significantly different between groups. The higher tertile of uric acid was, the lower eGFR was at 1 year post-transplant, representing an inverse relationship between uric acid levels and allograft function^11,27^. Biopsy-proven rejection was not significantly different but numerically higher in tertile 2 and tertile 3 than tertile 1 (46.3% vs. 56.8% vs. 65.0%, *P* = 0.057). Incidences of each diagnosis of biopsy were similar including CNI toxicity and polyoma virus nephropathy.

**Table 1:** Characteristics of transplant recipients by tertiles of uric acid level.

Variables	Tertile 1 (n = 80)	Tertile 2 (n = 81)	Tertile 3 (n = 80)	*P*
Age (years)	45.0 ± 10.8	40.4 ± 11.4	41.3 ± 12.9	0.031
Sex, males	51 (63.7%)	52 (64.2%)	51 (63.7%)	0.998
Body mass index, (kg/m^2^)	21.8 ± 2.9	22.9 ± 3.2	22.8 ± 3.3	0.046
Deceased donor	12 (15.0%)	19 (23.5%)	33 (41.3%)	0.001
Donor age (years)	42.6 ± 11.4	43.3 ± 10.9	48.8 ± 9.8	<0.001
Donor sex, males	33 (41.3%)	41 (50.6%)	38 (47.5%)	0.479
Pre-transplant diabetes mellitus	18 (22.5%)	13 (16.0%)	5 (6.3%)	0.015
Duration of pretransplant dialysis (months)	24.9 ± 37.9	29.1 ± 36.4	49.6 ± 53.8	0.001
Retransplantation	4 (5.0%)	5 (6.2%)	8 (10.0%)	0.434
Number of HLA mismatches	2.3 ± 1.2	2.6 ± 1.3	2.3 ± 1.2	0.172
Tacrolimus use	43 (53.8%)	50 (61.7%)	52 (65.0%)	0.327
Delayed graft function	6 (7.5%)	6 (7.4%)	15 (18.8%)	0.032
SBP at 1 year (mm Hg)	125.1 ± 12.5	125.5 ± 12.8	129.2 ± 13.3	0.085
DBP at 1 year (mm Hg)	80.1 ± 10.1	78.7 ± 9.4	81.1 ± 10.3	0.321
Use of ACE inhibitor	47 (58.8%)	48 (59.3%)	44 (55.0%)	0.837
Mean uric acid within the 1^st^ post-transplant year (mg/dL)	4.2 ± 0.6	5.5 ± 0.5	7.1 ± 1.0	<0.001
Use of uric acid lowering agent	4 (5.0%)	5 (6.2%)	7 (8.8%)	0.622
eGFR^a^ at 1 year (mL/min)	65.5 ± 21.6	58.4 ± 19.8	44.3 ± 18.3	<0.001
Interval between KT and biopsy (months)	23.7 ± 15.3	30.0 ± 18.6	27.5 ± 18.5	0.072
Diagnosis of biopsy				
Biopsy-proven rejection	37 (46.3%)	46 (56.8%)	52 (65.0%)	0.057
Calcineurin inhibitor toxicity	6 (7.5%)	5 (6.2%)	11 (13.8%)	0.205
Acute tubular injury	6 (7.5%)	5 (6.2%)	6 (7.5%)	0.930
Polyoma virus nephropathy	5 (6.3%)	4 (4.9%)	9 (11.3%)	0.276
Diabetic nephropathy	0 (0.0%)	0 (0.0%)	2 (2.5%)	0.131
IgA nephropathy	5 (6.3%)	5 (6.2%)	4 (5.0%)	0.931
Focal segmental glomerular sclerosis	1 (1.3%)	7 (8.6%)	3 (3.8%)	0.073
Immune complex-mediated glomerulonephritis, other than IgA nephropathy	0 (0.0%)	1 (1.2%)	1 (1.3%)	0.606

Data are mean ± standard deviation or number (%).

^a^Calculated using Chronic Kidney Disease Epidemiology (CKD-EPI) formula.

ACE, angiotensin converting enzyme; DBP, diastolic blood pressure; eGFR, estimated glomerular filtration rate; HLA, human leukocyte antigen; KT, kidney transplantation; SBP, systolic blood pressure.

### Banff pathologic score

**Figure 2:**
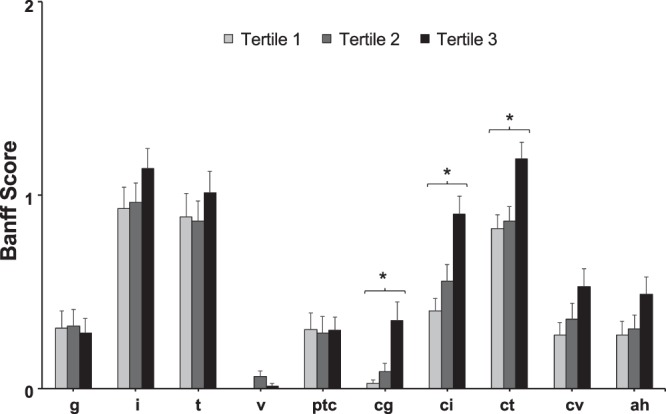
Comparison of mean values of Banff scores between uric acid tertiles. ^*^P < 0.05 by one way Analysis of Variance. ah, arteriolar hyalinosis; cg, glomerular basement membrane double contour; ci, interstitial fibrosis; ct, tubular atrophy; cv, vascular fibrous intimal thickening; g, glomerulitis; HighUA, high mean uric acid level; i, interstitial inflammation; ptc, peritubular capillaritis; t, tubulitis; v, intimal arteritis.

Figure. 2 shows comparisons for average of each pathologic (Banff) score. The ci and ct scores were significantly different between the groups (0.4 vs. 0.6 vs. 0.9, *P* < 0.001 and 0.8 vs. 0.9 vs. 1.2, *P* = 0.002, respectively), and cg score was also different (0.02 vs. 0.08 vs. 0.35, *P* = 0.001). In the post hoc analysis, those 3 chronic scores were significantly higher only in tertile 3 than other two groups (*P* < 0.001 and 0.010 for ci; *P* = 0.003 and 0.010 for ct; *P* = 0.001 and 0.009 for cg, vs. tertile 1 and tertile 2, respectively).

### Grade of interstitial fibrosis and tubular atrophy

We graded IF/TA from 0 to 3 according to the higher value of the ci and ct scores. There was a significant ordered relationship between the grade of IF/TA and uric acid level (*P* = 0.001) (Table 2). Overall severe IF/TA (ci ≥2 or ct ≥2) was 18.3% (n = 44). The higher uric acid tertile was, the more frequent severe IF/TA developed (8.8% [n = 7] vs. 19.8% [n = 16] vs. 26.3% [n = 21], *P* = 0.015). Although male patients had higher uric acid level than female, there was no difference in the severity of IF/TA between both sexes.

**Table 2:** Difference in the grade of IF/TA between uric acid tertiles.

IF/TA grade	All subjects (n = 241)	Tertile 1 (n = 80)	Tertile 2 (n = 81)	Tertile 3 (n = 80)	^*a*^ *P*
0	47 (19.5%)	19 (23.7%)	20 (24.7%)	8 (10.0%)	0.001
1	150 (62.2%)	54 (67.5%)	45 (55.5%)	51 (63.7%)	
2	33 (13.7%)	5 (6.3%)	14 (17.3%)	14 (17.5%)	
3	11 (4.6%)	2 (2.5%)	2 (2.5%)	7 (8.8%)	

^a^by Manteal-Haenzel chi square test.

IF/TA, interstitial fibrosis and tubular atrophy.

### Risk assessment for severe interstitial fibrosis and tubular atrophy

Univariate and multivariate analyses showed higher uric acid level was an independent risk factor for severe IF/TA (Table 3). Adjusted odds ratio (OR) of uric acid tertile 2 versus tertile 1 was 2.16 (95% confidence interval [CI] 1.13–8.82, *P* = 0.028) and that of tertile 3 versus tertile 1 was 3.70 (95% CI 1.25–10.93). Other independent determinants for severe IF/TA were eGFR at 1year post-transplant (per increase of 10 ml/min) (OR 0.8, 95% CI 0.65–0.98) and biopsy-proven rejection (OR 2.34, 95% CI 1.05–5.21). When considered as a continuous variable (per increase of 1 mg/dL), uric acid was still independent risk factor for severe IF/TA (OR 1.42, 95% CI 1.07–1.88).

**Table 3:** Risk factors associated with severe IF/TA.

Variables	Univariate		Multivariate^a^	
	OR (95% CI)	*P*	OR (95% CI)	*P*
Uric acid tertile				
Tertile 1	Reference		Reference	
Tertile 2	2.57 (0.99–6.63)	0.052	2.16 (1.13–8.82)	0.028
Tertile 3	3.71 (1.48–9.33)	0.005	3.70 (1.25–10.93)	0.018
Age, years	0.96 (0.93–1.01)	0.083		
Sex, male	0.55 (0.29–1.07)	0.078		
Body mass index	0.85 (0.76–0.96)	0.007	0.79 (220.69–1.30)	0.101
Deceased donor	1.37 (0.67–2.79)	0.383	0.83 (0.31–2.20)	0.702
Donor age, years	0.99 (0.96–1.02)	0.539	0.96 (0.93–1.01)	0.063
Retransplantation	0.96 (0.26–3.48)	0.946		
Delayed graft function	2.56 (1.06–6.16)	0.036	1.55 (0.46–5.25)	0.479
SBP at 1year (every 10 mmHg)	1.07 (0.94–1.38)	0.578		
Use of ACE inhibitor	0.77 (0.40–1.47)	0.423		
Use of uric acid lowering agent	1.54 (0.47–5.03)	0.473		
eGFR at 1year (every 10 ml/min)	0.81 (0.69–0.96)	0.014	0.80 (0.65–0.98)	0.036
Biopsy-proven rejection	2.79 (1.34–5.84)	0.006	2.34 (1.05–5.21)	0.037

^a^Multivariate analysis was performed by logistic regression.

ACE, angiotensin converting enzyme; CI, confidence interval; eGFR, estimated glomerular filtration rate; IF/TA, interstitial fibrosis and tubular atrophy; KT, kidney transplantation; OR, Odds ratio; SBP, systolic blood pressure.

### Graft survival

**Figure 3:**
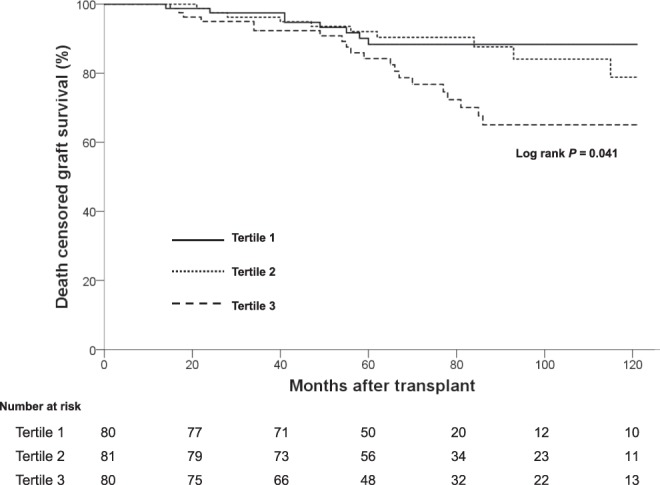
Kaplan-Meier survival estimates for death censored graft survival over 10 years.

The Kaplan-Meier survival curves showed that death censored graft survival over 10 years was significantly different between three groups (*P* = 0.041) (Fig. 3). In the post hoc analysis, only tertile 3 had significantly lower survival than tertile 1 (*P* = 0.036) or tertile 2 (*P* = 0.041).

## Discussion

By comparing Banff pathologic scores from renal biopsies, we demonstrated that higher uric acid was associated with more severe fibrosis in transplanted kidneys. Several experimental studies have shown evidence implicating uric acid in the progression of fibrosis^28^. Moreover, a recent study hypothesized molecular mechanism of renal injury from soluble uric acid by upregulation of NALP3 inflammasome^29^ which has been suggested as an endogenous pathway of tissue injury in other organs such as lung^30^ as well as gout^31,32^. However, it has been debated whether hyperuricemia exerts a causal effect on the deterioration of renal allografts and native kidneys with chronic disease.

Because serum uric acid and renal function bidirectionally interact with each other, the resultant decline in eGFR could mask the effect of uric acid itself, thus necessitating a longitudinal approach examining time-varying eGFR and uric acid levels. In a retrospective analysis, Kim *et al*.^10^ concluded that uric acid was not an independent risk factor for renal allograft loss. However, another large cohort study, using a similar analytical method but a longer follow-up period, showed that uric acid was a risk factor^7^. Additionally, Tsai *et al*.^33^ reported that higher uric acid levels were independently associated with end-stage renal disease and all-cause mortality in patients with chronic kidney disease (CKD) based on the concept of trajectories of uric acid and corresponding eGFRs. Even in the Korean population, where the prevalence of gout is low as 0.4%^34^, there was a report that higher serum uric acid was associated with increased risk of CKD^35^. Demonstration of the relationship between uric acid and graft loss or progression of CKD requires long-term follow-up. Therefore, we hypothesized that estimating the differences in chronic renal histologic injuries would help distinguish the true effects of uric acid.

Based on analysis of surveillance biopsies, Stegall *et al*.^36^ demonstrated that the majority of renal allografts functioning over 10 years contained chronic injury. They also alluded to the necessity for intensive management of metabolic factors. Another study reported that rejection or the underlying presence of donor-specific antibody (DSA) might be major determinants of fibrosis^25^. However, treatment benefits after subclinical rejection have so far been restricted to low-risk recipients and have not yet been established in cases of long-term graft survival^26^, indicating that immunologic causes are not the only factors deserving attention. From this perspective, uric acid could be one of the primary concerns in preventing allograft fibrosis.

Few studies have described the relationship between uric acid and allograft fibrosis. Alkalin *et al*. reported that hyperuricemia was associated with chronic allograft nephropathy (CAN)^37^, but that terminology is now outdated and, more specifically, it includes rejection and nonspecific tubulointerstitial fibrosis. Furthermore, hyperuricemia was an independent risk factor for pooled outcomes including death, graft loss, and CAN, but not for CAN alone. More recently, Hart *et al*.^38^ demonstrated that hyperuricemia was a risk factor for doubling of the interstitium or end– stage renal disease from IF/TA after KT in a post hoc analysis of a prospective study. However, this study involved a small study population and the multivariate analysis endpoint was intermixed with graft losses from all other causes. Therefore, it was not clear whether hyperuricemia independently increased the risk of IF/TA. Additionally, neither of those two studies compared pathologic scores or considered the underlying pathologic diagnoses. Our results provided more definitive information about the relationship between uric acid level and allograft fibrosis by analyzing pathologic scores as endpoints, especially in the presence of known risk factors such as corresponding renal function and rejection.

In a Korean population study, they showed early post-transplant hyperuricemia was an independent risk factor for allograft loss^7^. In contrast, we divided KT patients according to sex specific tertiles of serum uric acid and showed an ordered relationship between uric acid level and IF/TA; even tertile 1 showed less severe IF/TA than tertile 2 of which uric acid level was conventionally normal. This result could provide not only a pathologic evidence to support previous studies about uric acid and allograft outcome^4,5,7,8^ but also an inspiration for the future study on lowering uric acid below the normal level to reduce allograft fibrosis.

In a recent review, Vanhove *et al*. summarized several therapeutic approaches to reducing the progression of allograft fibrosis after KT, such as CNI-sparing immunosuppression, reduction of renin-angiotensin-aldosterone system activation, appropriate management of subclinical rejection, and direct intervention for fibrogenic molecules^26^. No interventional study has examined the effects of hyperuricemia treatment on renal fibrosis, but many studies have examined the effects of uric acid-lowering agents on eGFR decline in patients with CKD^39^. These include a well-designed randomized controlled trial using febuxostat, which is a newly developed xanthine oxidase inhibitor^40^. In the transplantation field, only a few retrospective, small-volume studies have evaluated the effects of lowering uric acid levels on renal allograft outcome^41–43^. The extent to which hyperuricemia contributes to fibrogenesis of an allograft has not been fully established, especially in the presence of immunologic challenges; however, our study shows that lowering uric acid levels could be an antifibrotic option.

Our study has limitations. As reported in a recent large cohort study, circulating DSA is one of the main contributors to allograft fibrosis^25^, but it was not assessed in this study because laboratory detection of DSA has been available in our institution since only 2011. Also, because renal biopsies are not standard parts of the post-transplant protocols in our institution, the biopsies included in this study were performed only when deemed indicated by the clinical care team. So, we could not show the changes over time in pathologic scores representing graft fibrosis. A study with routine serial biopsies is necessary to provide more information about the effect of higher uric acid on fibrosis progression.

Despite these limitations, our study demonstrated that higher uric acid level was an independent risk factor for severe IF/TA after kidney transplantation, even in the presence of corresponding renal allograft function and rejection. The results would be not only a good help for the clinical decision about lowering serum uric acid after kidney transplantation but also inspiration for the future research on the allograft fibrosis and long-term outcome.

## Methods

### Materials

**Figure 4:**
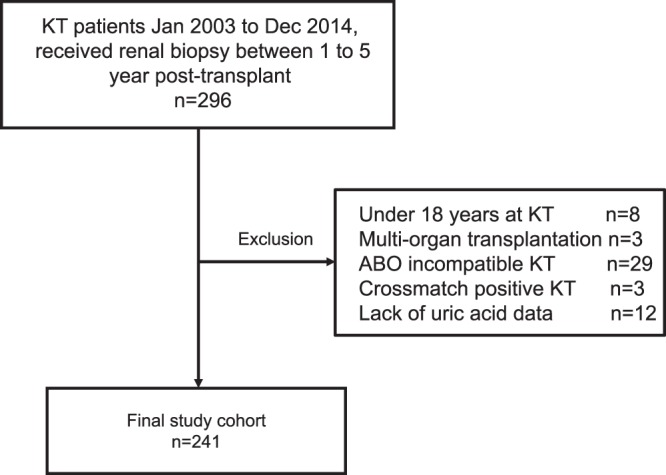
Study population. KT, kidney transplantation.

We conducted a retrospective analysis of prospectively collected data from 296 patients who underwent KT from January 2003 to December 2014 and received renal biopsy during 1 to 5 post-transplant year. The exclusion criteria were as follows: (1) age <18 years at the time of KT, (2) multi-organ transplantation, (3) ABO-incompatible KTs, (4) crossmatch-positive KTs, and (5) insufficient uric acid data (Fig. 4). Finally, 241 patients were analyzed in this study. The biopsies were performed in our study population solely when clinically indicated, such as for increases in serum creatinine or increases in proteinuria. Biopsies performed at the time of surgery were not considered.

### Data collection

We obtained serum uric acid levels at 1, 3, 6, 9, and 12 month post-transplant. Patients were divided into three groups according to the sex specific tertiles of their mean uric acid level within the first year. Characteristics of the recipients and donors were also collected from the database.

### Pathologic review

All biopsies performed between 1 and 5 year post-transplant were examined by experienced renal pathologists (HJJ, BJL) and scored for glomerulitis (g), interstitial inflammation (i), tubulitis (t), intimal arteritis (v), peritubular capillaritis (ptc), glomerular basement membrane double contour (cg), interstitial fibrosis (ci), tubular atrophy (ct), vascular fibrous intimal thickening (cv), and arteriolar hyalinosis (ah)^44^. In case of multiple results of biopsy during study period, highest value of each score were recorded. Interstitial fibrosis and tubular atrophy are known to consistently occur together^45^, so IF/TA was graded according to the higher of the ci score or ct score.

We reviewed the pathology reports diagnosed by criteria before the 2007 Banff meeting report^46^ so that rejection diagnosis could be determined using the 2007 diagnostic criteria. Other pathologic diagnoses were confirmed in each biopsy, including CNI toxicity, acute tubular injury, polyomavirus nephropathy, diabetic nephropathy, IgA nephropathy, focal segmental glomerulosclerosis, and immune complex-mediated glomerulonephritis other than IgA nephropathy.

### Study endpoints

Our primary endpoint was incidence of severe IF/TA (ci ≥ 2 or ct ≥ 2)^25^. We also investigated the difference in death censored graft survival between groups.

### Statistical analysis

Data are shown as mean ± standard deviation for continuous variables and number (frequency) for categorical variables. The one-way Analysis of Variance and chi square test were used when appropriate. To examine ordered-relationship between uric acid tertile and degree of IF/TA, the Mantel-Haenszel chi square test was performed. Logistic regression was utilized in the univariate and multivariate analyses to determine whether uric acids levels independently affected allograft fibrosis. Considering the sizes of the study populations, only covariates with a *P* value < 0.05 in the univariate analysis and known risk factors of allograft fibrosis in the literature were entered into the multivariate analysis. Univariate and multivariate results were reported as odds ratios (ORs) with 95% confidence intervals (CIs). Kaplan-Meier survival curves were compared using the log rank test. All analyses were performed using SPSS software (version 23.0; SPSS, Inc., Chicago, IL, USA) and *P* values < 0.05 were considered statistically significant.

### Ethic statement

The study was conducted according to the principles of the Declaration of Helsinki and approved by the independent Institutional Review Board of Yonsei University College of Medicine (IRB No.: 4-2017-1154).
